# Individual Differences in Anticipatory Somatosensory Cortex Activity for Shock is Positively Related with Trait Anxiety and Multisensory Integration

**DOI:** 10.3390/brainsci6010002

**Published:** 2016-01-06

**Authors:** Steven G. Greening, Tae-Ho Lee, Mara Mather

**Affiliations:** 1 Department of Psychology, Louisiana State University, Baton Rouge, LA 70803, USA; 2 Department of Psychology, University of Southern California, Los Angeles, CA 90089, USA; taehol@usc.edu; 3 Davis School of Gerontology, University of Southern California, Los Angeles, CA 90089, USA; 4 Neuroscience Graduate Programs, University of Southern California, Los Angeles, CA 90089, USA

**Keywords:** anxiety, fear, fear conditioning, functional connectivity, fear network, multisensory integration, emotion, fMRI, amygdala

## Abstract

Anxiety is associated with an exaggerated expectancy of harm, including overestimation of how likely a conditioned stimulus (CS+) predicts a harmful unconditioned stimulus (US). In the current study we tested whether anxiety-associated expectancy of harm increases primary sensory cortex (S1) activity on non-reinforced (*i.e.*, no shock) CS+ trials. Twenty healthy volunteers completed a differential-tone trace conditioning task while undergoing fMRI, with shock delivered to the left hand. We found a positive correlation between trait anxiety and activity in right, but not left, S1 during CS+ *versus* CS− conditions. Right S1 activity also correlated with individual differences in both primary auditory cortices (A1) and amygdala activity. Lastly, a seed-based functional connectivity analysis demonstrated that trial-wise S1 activity was positively correlated with regions of dorsolateral prefrontal cortex (dlPFC), suggesting that higher-order cognitive processes contribute to the anticipatory sensory reactivity. Our findings indicate that individual differences in trait anxiety relate to anticipatory reactivity for the US during associative learning. This anticipatory reactivity is also integrated along with emotion-related sensory signals into a brain network implicated in fear-conditioned responding.

## 1. Introduction

A core feature of anxiety involves disabling thoughts focused on the anticipation and expectations of harm or danger [1]. Fear-related associative learning, or fear conditioning, is a widely used paradigm for studying anxiety-related processes. In differential fear conditioning, an otherwise benign stimulus, such as a tone (conditioned stimulus, CS+), is repeatedly paired with an aversive stimulus, such as a mild shock (unconditioned stimulus, US), while a second tone is never paired with shock (CS−). In this manner, presentation of the CS+ produces a conditioned response (CR) similar to the unconditioned response (UR) produced naturally by the US (e.g., increased sweating elevates skin conductance response, or SCR). Both explicit participant reports of US expectancy and implicit differential electrodermal activity, including SCR, are robust indices of US anticipation for perceptible conditioned stimuli [2,3,4]. Furthermore, individuals high in trait anxiety overestimate the likelihood that the CS+ will be paired with the US [4,5].

Previous neuroimaging studies of fear conditioning, or associative learning more generally, have focused on the role of the amygdala in the formation of CS+/US associations [6,7,8], or interactions between the amygdala and insula in threat anticipation [9]. However, in addition to the amygdala, others have emphasized the importance of more complex, higher-order processing in accurate fear conditioning [10]. Higher-order processes, like perception and executive attention, and related regions, such as early sensory cortices [11,12,13,14] and frontoparietal cortices [15,16,17], are involved in more complex forms of fear conditioning, like the differential fear conditioning example provided above as compared to single-cue conditioning, in which there is no CS−. In terms of the sensory cortices corresponding to the conditioned stimuli, greater activation for the CS+ *versus* the CS− has been observed in the visual [18,19], the auditory [20,21] and the olfactory [22] cortices in human neuroimaging studies. Moreover, the generalization of conditioned fear for visual conditioned stimuli appears to involve interactions between the amygdala and the extrastriate visual cortex [23,24]. The frontoparietal regions, in particular the dorsolateral prefrontal cortex (dlPFC), also contribute to the acquisition and the formation of accurate representation of the learned association between conditioned and unconditioned stimuli [25,26]. For example, Carter *et al.* (2006) demonstrated that during both delay and trace conditioning, dlPFC activity reflects the degree of explicit contingency knowledge for the CS+ regardless of whether the trials were reinforced or non-reinforced [15].

One intriguing aspect of the contingency formation in fear conditioning is that it can involve the integration of information across multiple senses. Although often overlooked in the human neuroimaging literature, evidence from both non-human animal [13] and human [27] research has demonstrated that the sensory cortices corresponding to both the US (*i.e.*, primary somatosensory cortex, S1, in the case of shock-based conditioning) and the CS (*i.e.*, primary auditory cortex, A1, in the case of auditory tones) are active during the acquisition phase of fear conditioning. Most notably, using electroencephalography (EEG), Miltner *et al.* (1999) demonstrated that fear conditioning using visual conditioned stimuli and mild electrical shock to the finger produced significantly greater gamma band coherence between the visual and somatosensory cortex in the hemisphere contralateral to the hand being shocked [27]. They confirmed their findings using both shock to the left hand and shock to the right hand in two independent samples of participants. Given the limitations of EEG, Miltner *et al.* were unable to also record activity from the amygdala. Using fMRI, at least two studies have similarly found that during conditioning there is greater activity in the primary sensory cortex associated with the US [18,28]. Importantly, these observations of US-related sensory activity are from trials in which no reinforcement is present to confound subsequent brain activity. Despite these earlier findings, little research to date has focused on the importance of US sensory cortex activity in fear conditioning on non-reinforced trials (*i.e.*, US-related brain activity). Furthermore, it is unknown how such a representation becomes integrated into a multisensory fear network. Given the expectancy biases observed in high anxious individuals, one possibility is that trait anxiety is positively related to individual differences in sensory cortex activity corresponding to the US. An additional possibility is that fear conditioning involves a process of multisensory integration in which the CS triggers mental representations of the US that engage sensory cortex because the sensation of the shock is recalled and also activates the amygdala due to the emotional significance of the associated US. Such integration, furthermore, should reflect individual differences and it may also rely on contributions from higher-order attention-related regions including dlPFC, as noted above [15].

While the notion of multisensory integration tends to be implicit in associative learning paradigms, the role and importance of multisensory integration has not received much focus in the human neuroimaging literature [29]. Moreover, a systematic review [29] found that the studies that reported sensory cortex activity primarily focused on the sensory cortex activity corresponding to the CS, not the US. Fewer still consider correlations between the sensory cortices of the CS and US.

The purpose of the present study was to determine whether US-related brain activity in the sensory cortex during the anticipation of shock was associated with anxiety. Single subject shock anticipation was represented as the amount of right S1 activity during non-reinforced CS+ trials compared to CS− trials. We also confirmed implicit anticipation of the US independently with the SCR data. We combined a differential tone-based (CS) trace conditioning paradigm, in which mild shock was used as the US, with a region-of-interest (ROI) based functional magnetic resonance imaging (fMRI) approach. We first tested the prediction that individual differences in trait anxiety positively correlate with greater right, but not left, differential primary somatosensory cortex (S1) activity on non-reinforced (*i.e.*, no shock) CS+ compared to CS− trials. (We examined right S1 because shock was exclusively delivered to the left hand of participants.) Second, we tested the prediction that right S1 activity is integrated into a fear-related network involving the amygdala and the primary sensory cortex of the CS (*i.e.*, the primary auditory cortex, A1, given the use of tones as the CS). To do this, we examined whether individual differences in brain activity related to shock anticipation in right, but not left, S1 were correlated with activity in both A1 and the amygdala. Finally, we used a functional connectivity analysis to test the prediction that trial-by-trial activity in S1 would correlate with activity in attention-related regions, such as dlPFC, reflecting the role of attention in contingency awareness. To address these aims, the current study examined the blood-oxygenation-level-dependent (BOLD) response during a fear conditioning session. Basic whole-brain findings from an examination of the entire trial (tone plus trace interval) were previously reported as supplemental information for an independent study focusing on a second, post-conditioning phase of the study [30].

## 2. Experimental Section

### 2.1. Participants

Twenty healthy participants (11 female) with mean age of 21.95 (range 18–35) participated in the study. All participants completed the Spielberger State-Trait Anxiety Inventory (STAI, [31]) before the fMRI scan. The STAI is an established measure of individual differences in anxiety, which has also been widely used in the fMRI literature to measure the relationship between brain activity and individual differences in anxiety [32,33,34,35]. Participants’ mean state anxiety (STAI-S) score was 29.50 (range 22–42, SD = 6.33), and their mean trait anxiety (STAI-T) score was 32.40 (range 21–50, SD = 7.00). Standard MRI inclusion criteria were applied for participant recruitment, such that they had to be safe for participation in the MRI environment. In addition, our *a priori* exclusion criteria included participants who self-reported having a clinically diagnosed mental illness or who reported being on, or having taken in the past, any pharmacological intervention for a mental illness. However, no participants fitting these criteria volunteered for participation in the study, nor were any participants excluded from the analysis *post hoc* due to issues of data quality, e.g., excessive motion artifacts. All participants gave informed consent in accordance with University of Southern California Institutional Review Board guidelines.

### 2.2. Design and Procedure

Stimuli and apparatus. Two tones (500 and 1500 Hz) were adopted as conditioned stimuli (CSs). The schedule of stimulus presentation and data collection were controlled by the PsychToolbox extensions [36,37] based on Matlab 2010b (MathWorks Corp., Natrick, MA, USA). The mild electric shock used as an unconditioned stimulus (US) was delivered to the third and fourth fingers of the left hand via a shock stimulator (E13–E22; Coulbourn Instruments, Allentown, PA, USA), which included a grounded RF filter. The intensity of “highly unpleasant but not painful” electric shock was determined individually (mean intensity = 2.30 mA, range 1.4–4.0 mA). Trials that included shocks were excluded in subsequent analyses.

**Figure 1:**
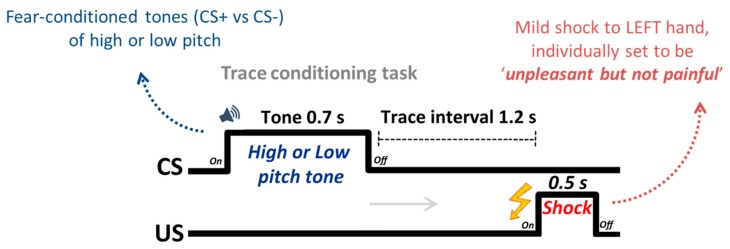
Trial structure for the fear conditioning task. A total of 30 trials were presented in a random order: 10 CS+ with shock, 10 CS+ without shock and 10 CS− tones. Thus, CS+ tones were followed by a shock with a 50% partial reinforcement schedule.

Fear-conditioning task (see Figure 1). The conditioning task consisted of one run in which either the low- or high-pitched tone was paired with electric shock. Which tone was paired with shock was counterbalanced across participants; eleven participants were conditioned with the high-pitched tone and 10 with the low-pitched tone as the CS+. Each trial in the conditioning session began with the onset of a fixation cross against a gray background. Participants were then presented with one of the CS tones for 0.7 s, followed by a 1.2 s inter-stimulus interval. After this interval, a shock was delivered for 0.5 s if the tone was assigned to the CS+ condition and followed by a fixation jittered to appear for 10, 11 or 12 s. On the CS− tone trials, there was no shock. To ensure some ambiguity between the CS−US contingency ratio, participants were only required to perform a tone (CS) discrimination task. They were asked to indicate by button press with their index or middle finger of the right hand whether the tone was low or high pitched immediately after hearing the tone. In this manner, they were not explicitly engaged in trying to disambiguate the contingency ratio. Participants were, however, informed of which CS would be paired with shock prior to study commencement, consistent with a number of fear conditioning studies [29,38]. Fear contingent responding was assessed using skin conductance response (SCR). Although trace conditioning is a weaker form of associative learning, its use ensured that any activity in S1 would be due to the expectation and forecasting of threat, as even on reinforced trials (which were regressed out of the main analysis) shock did not temporally overlap with CS presentation. It also extends previous findings of multisensory integration during delay conditioning from the EEG literature [27]. A total of 30 trials were presented in a random order: 10 CS+ with shock, 10 CS+ without shock and 10 CS− tones. Thus, CS+ tones were followed by a shock with a 50% partial reinforcement schedule. This approach was successful at eliciting a conditioned response on non-reinforced CS+ trials compared to CS− trials, as reported previously [30]. The imaging session also involved a separate task following the conditioning phase, the details of this independent task were described previously by Lee *et al.* (2014) [30].

### 2.3. Physiological Recordings

As described previously [30], individual skin conductance responses (SCRs) were acquired to confirm the success of the emotional arousal manipulation with MRI-compatible electrodes placed on the index and middle finger of the left hand. All physiological data were recorded at 1 kHz sampling rates through the MP-150 system (BIOPAC System, Goleta, CA, USA), connected to a grounded RF filter, and MR-compatible leads and electrodes. The SCR data were detrended and smoothed with a median filter over 50 samples to filter out MRI-induced noise for offline analysis. SCR data epochs were extracted from a time window between 0 and 8 s after CS tone onset, and baseline-corrected between 0 and 1 s. The peak SCR amplitude from baseline was taken between 1–8 s on a trial-by-trial basis then averaged as a function of CS tone. Due to a technical failure, recording could not be completed for one participant.

### 2.4. MRI Data Acquisition

All MRI data were acquired on a Siemens 3T Magnetom Trio with a liquid crystal display projector (1024 × 768 pixels at 60 Hz) onto a rear project screen behind the head of participants and viewed using a mirror attached to a 32-channel matrix head coil at the University of Southern California Dana and David Dornsife Cognitive Neuroscience Imaging Center. High-resolution (T1-MPRAGE) structural images were acquired first (repetition time or TR = 1950 ms; echo time or TE = 2.26 ms; flip angle or FA = 7°; 1 mm isotropic voxel; 256 mm field of view). Next, functional images were acquired with gradient-echo echo-planar T2*-weighted imaging. Each functional volume consisted of 40 interleaved (no skip) 2.5 mm axial T2*-weighted slices (TR = 2000 ms; TE = 25 ms; FA = 90°; matrix size = 64 × 64; field of view = 192 mm).

### 2.5. General fMRI Data Analysis

*Preprocessing*: Standard preprocessing was conducted using FMRIBs Software Library (FSL); slice-time correction, motion correction with MCFLIRT, spatial smoothing with a Gaussian kernel of full width at half maximum (FWHM) 6 mm, high-pass temporal filtering with a filter width of 100 s and skull stripping of structural images with FSL’s rain Extraction Tool (BET), and registering each functional image to both the participant’s high-resolution structural image and the standard Montreal Neurological Institute (MNI) 2 mm brain. MELODIC ICA2 [39] was applied to remove noise components. The first eight volumes were discarded to allow for signal equilibration.

Primary general linear model (GLM-1): As our main interests were how somatosensory processing during fear conditioning is modulated by trait anxiety during the perceptual processing and disambiguation of conditioned stimuli, and how such multisensory information is integrated, we estimated individuals’ BOLD signal during the CS tone interval. In this way we produced two critical regressors. The first was for non-reinforced CS+ trials and the second was for CS− trials. To do so we used a gamma-variate function plus temporal derivative. Additionally, six motion parameters and shock-reinforced trials were modeled as covariates of no interest. Basic whole-brain findings from an examination of the entire trial (tone plus trace interval) were previously reported as supplemental information in Lee *et al.*’s work [30]. Although the main goal of this study was to investigate the relationship between individuals’ anxiety level and the somatosensory processing, a whole-brain group-level random-effects analysis was also performed to identify general brain activation at a group level. Group level analysis was thresholded using cluster detection statistics, with a height threshold of *Z* > 2.3 cluster corrected to *p* < 0.05 (one-tailed) [40].Whole-brain multiple comparisons correction was carried out using Gaussian Random Field Theory.

Secondary general linear model (GLM-2): We ran a secondary GLM to confirm that mild shock per se to the left hand produced greater activity in right and not left somatosensory cortex. This involved modelling the shock epoch on reinforced CS+ (*i.e.*, shocked) trials (500 ms), using the- same temporal epoch for both non-reinforced CS+ and CS− trials. The results of GLM-2 were analyzed using the anatomical ROIs of right and left S1.

### 2.6. General Region of Interest Analysis and Correlation

**Figure 2:**
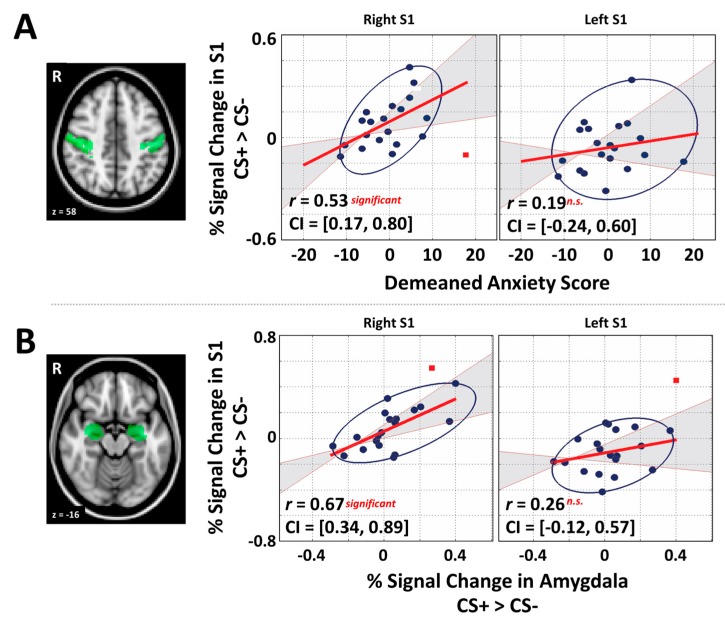
(**A**) The anatomical structural masks of S1 used in this study and scatter plots illustrating the correlation between individual trait anxiety level and evoked signal in the S1 ROI of the right and left hemispheres, respectively; (**B**) the anatomical structural masks of amygdala (bilateral) and scatter plots illustrating the correlation between evoked signal in the amygdala and the S1 region. Gray-shaded area indicates 95% bootstrapped CIs. Red square data points indicate bivariate outliers that were identified by the robust method [44].

The first goal of the current study was to assess the relationship between individual anxiety and responses in the right S1 (*i.e.*, region corresponding to shock delivery, see Figure 2A). Accordingly, percent signal change values were extracted individually using FSL Featquery within the right S1 region for each CS tone. As a control, the left S1 (*i.e.*, non-shock related region) was also investigated. These left and right S1 masks were obtained from a standard anatomical brain atlas provided by FSL (Jülich histological atlas). Additionally, bilateral amygdala (see Figure 2B) and auditory regions (A1) were also investigated with bilateral anatomical masks obtained from the same database. Use of independent anatomical ROI masks allow for the unbiased calculation of effect size estimates by avoiding circularity [41,42]. Initial individual differences analyses between STAI-T, S1, A1, and amygdala activity were carried out using the robust correlation method [43]. The robust method was used as it identifies bivariate outliers and removes them in the calculation of a 95% confidence interval (CI) [44].

### 2.7. Mediation Analysis

A standard mediation analysis approach was adopted at the group level in order to delineate the relationship between our ROIs. This involved evaluating the following components using the mediation toolbox [45]: (1) the total effect *c* (initial predictor variable → outcome, which can also be written in terms of the indirect effect *abs* + the direct effect *c’*); (2) the indirect path *a* (initial mediation analysis variable → mediator variable); (3) the indirect path *b* (mediator variable → outcome after controlling for the initial variable); (4) the direct effect *c’* after controlling for the influence of the indirect path (initial predictor variable → outcome after controlling for the mediation effect); and (5) the mediation effect *abs*, which is the product of *ab*.

### 2.8. Functional Connectivity Analysis

To characterize dynamic interregional interactions between brain regions, a beta series correlation analysis [46,47] was applied. To do so, a new design matrix was created where unique trial-by-trial regressors were created for each of our conditions of interest (*i.e.*, CS+ no shock, and CS− trials). This resulted in 20 independent variables (*i.e.*, 10 CS+ tones and 10 CS− tones). The global mean signal level over all brain voxels was calculated for each time point and was included to reduce the confounding effects of the global signal change. Motion parameters and shock trials were also included in the design matrix as covariates of no interest. Finally, mean parameter estimates for each trial from the seed region (*i.e.*, S1) were extracted and used to compute correlations between the seed’s signal and signal of all other voxels in the brain, thus generating condition-specific seed correlation maps. Correlation magnitudes were converted into *z* scores using the Fisher’s *r*-to-*z* transformation. Condition-dependent changes in functional connectivity were assessed using random effects analyses, which were thresholded at the whole-brain level using clusters determined by *Z* > 2.3 and a cluster significance threshold of *p* = 0.05 (*corrected*; one-tailed).

## 3. Results

### 3.1. Physiological Index of Expectancy

The success of the current fear conditioning study was confirmed by the fact that CS+ trials yielded greater SCRs than CS− trials (*t*(18) = 2.20, *p* < 0.05). This also indicates an expectation of threat.

### 3.2. ROI Univariate Effects

GLM-1: First we examined whether there were univariate differences between the CS+ and CS− conditions in our ROIs, by comparing the parameter estimates for each condition. Whereas in the right S1 we observed marginally significant activity in the CS+ compared to the CS− (M_CS+_(SEM) = 0.18(0.04), M_CS−_(SEM) = 0.10(0.04), *p* = 0.055, two-tailed), in left S1 we observed significantly greater activity in the CS− compared to the CS+ condition (M_CS+_(SEM) = 0.29(0.06), M_CS−_(SEM) = 0.39(0.05), *p* < 0.05, two-tailed). In the bilateral A1 ROI we also observed significantly greater activity in the CS+ *versus* CS− condition (M_CS+_(SEM) = 0.36(0.04), M_CS−_(SEM) = 0.24(0.05), *p* < 0.005, two-tailed). However, there was no significant difference between the CS+ and CS− conditions in the amygdala (*p* > 0.4). Although we observed only a marginally significant univariate effect between the CS+ and CS− in right S1, this analysis is orthogonal to the individual differences approach that is the focus of the current study. Indeed, a number of papers have found individual differences in brain activity despite an absence of univariate effects [48,49,50].

GLM-2: The secondary GLM, which modeled the shock epoch of the trial, confirmed that mild shock to the left hand produced significantly greater activity in right S1 and not left S1. In right S1 there was significantly greater activity on reinforced (shocked) CS+ compared to non-reinforced CS+ trials (*t*(19) = 5.75, *p* < 0.001, two-tailed), and significantly greater activity on reinforced (shocked) CS+ compared to CS− (*t*(19) = 6.24, *p* < 0.001, two-tailed). Additionally, as expected given the lack of stimulation to the right hand, in left S1 there was no significant differences for reinforced CS+ compared to either non-reinforced CS+ trials (*t*(19) = 1.79, *p* = 0.09) and CS− trials (*t*(19) = −0.33, *p* = 0.74).

### 3.3. Primary Somatosensory Cortex and Individual Differences Analyses

To perform individual-difference (*i.e.*, correlation) analyses we subtracted the percentage signal change value for CS− from that of CS+ in all ROIs (*i.e.*, S1 for each hemisphere, bilateral A1 and bilateral amygdala), which provided an index of fear-induced reactivity for the CS+ relative to CS−. As predicted, a significant positive correlation between right S1 and individual trait anxiety (STAI-T) was observed, robust Pearson *r* = 0.53, *p* < 0.05, 95% CI after bootstrapping: [0.17, 0.80], indicating that increased somatosensory activity in the region responsible for processing the shock sensations from the left hand was associated with increased anxiety (Figure 2A). Moreover, as predicted, somatosensory responses in left S1 (associated with the non-shocked right hand) did not show any relationships with STAI-T (robust Pearson *r* = 0.19, *p* > 0.05, CIs: [0.24, 0.60] Figure 2A).

We next tested the prediction that right, and not left, S1 activity would correlate with activity in both the amygdala and A1. As predicted, this analysis revealed that right S1 showed a significant positive relationship with amygdala activation, robust Pearson *r* = 0.67, *p* < 0.05, 95% CI after bootstrapping: [0.34, 0.89] (Figure 2B). Left S1 showed no significant correlation with the amygdala (robust Pearson *r* = 0.26, *p* > 0.05, CIs: [0.12, 0.57] Figure 2B). We also found that A1 showed a significant correlation with right S1 as predicted (robust Pearson *r* = 0.71, *p* < 0.05, 95% CI after bootstrapping: [0.31, 0.91]); however, A1 also correlated with left S1 (robust Pearson *r* = 0.64, *p* < 0.05, 95% CI after bootstrapping: [0.37, 0.85]), which might reflect the association between the tones and behavioral responding with the right hand. Additionally, there was a strong relationship between the amygdala and A1 regions (robust Pearson *r* = 0.63, *p* < 0.05, 95% CI after bootstrapping: [0.29, 0.84]). Lastly, although previous research using fear conditioning found that STAI-T positively correlates with fear-induced amygdala reactivity [35], we found no such correlation (*r* = 0.0132, *p* > 0.05, CIs: [−0.51, 0.65]).

### 3.4. Mediation Analysis

**Figure 3:**
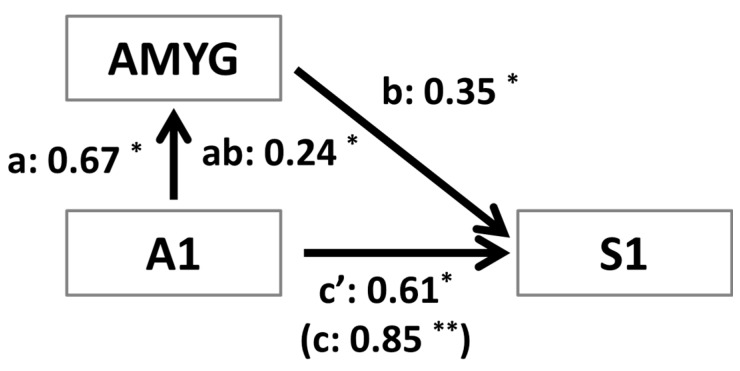
Mediation analysis at the group-level using the mean responses in each region per participant. For all variables (*i.e.*, regions) in the network, signal differences between CS+ and CS− were used as the dependent measure. Path analysis was used to test the hypothesis that the initial effect of primary auditory reactivity (A1) on the response in the primary sensory cortex (S1) was mediated by reactivity of the amygdala (AMYG). As predicted, we observed a significant mediation effect (*i.e.*, *abs* = 0.24), and the coefficient of the *c’*_1_ path (*i.e.*, the effect of A1 on S1 after controlling for the amygdala as a mediator) was diminished yet still significant. The latter suggests A1 activity had an influence of S1 activity even after controlling for the amygdala mediation. * *p* < 0.05; ** *p* < 0.01.

To further investigate the correlation results showing that right S1 is significantly correlated with A1 and amygdala activity, we performed a mediation analysis. We set up the mediation analysis in a manner consistent with the apparent flow of information present in our experimental design. We tested the hypothesis that the effect of A1, which was the region corresponding to the initial sensory input (*i.e.*, the tones), on right S1 activity was mediated by amygdala reactivity. As shown in Figure 3, the path between A1 and amygdala (path *a*_1_; *a*_1_ = 0.67, *p* < 0.05) and the path between amygdala and right S1 (path *b*_1_; *b*_1_ = 0.35, *p* < 0.05) were statistically significant. Importantly, the mediation effect was also significant (*ab*_1_ = 0.24, *p* < 0.05) indicating that the amygdala mediated the observation of enhanced activity in right S1 during the CS+ *versus* CS− condition. Additionally, even after controlling for the mediation effect, A1 had a significant influence on S1 activity.

### 3.5. Functional Connectivity Analysis

**Figure 4:**
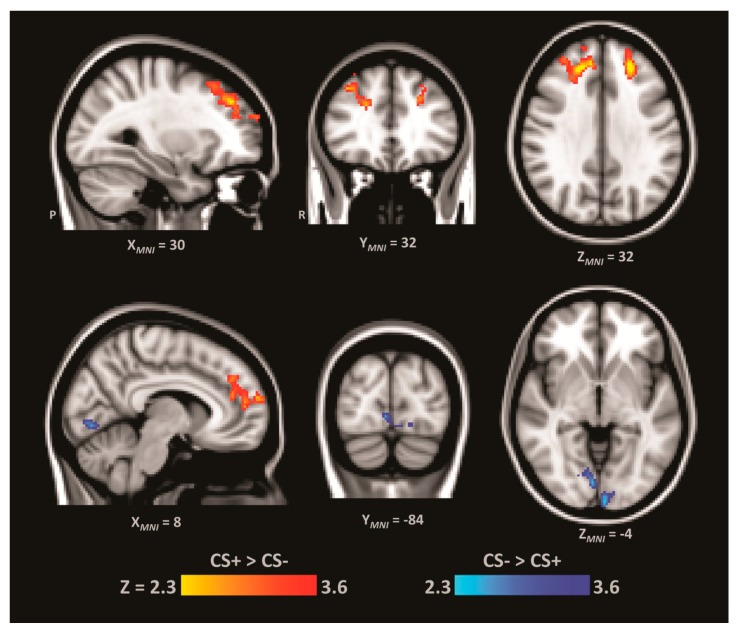
Brain regions showing both greater connectivity with S1 during CS+ than CS− (RED–YELLOW) and less connectivity with S1 during CS+ than CS− (BLUE–LIGHT BLUE). This reveals increased connectivity during CS+ trials between right S1 and bilateral dlPFC and dmPFC. It also reveals decreased connectivity during CS+ trials between right S1 and parts of lingual gyrus.

Finally, we sought to test the prediction that trial-by-trial activity in right S1 is functionally correlated with activity in regions implicated in higher-order cognitive processes. To do so, we performed a whole-brain connectivity analysis comparing the CS+ and CS− trials (Figure 4; for full results see Table 1). This analysis revealed that right S1 (*i.e.*, seed region) had greater positive functional connectivity with prefrontal regions including the dlPFC (*i.e.*, middle frontal gyrus) and the dorsomedial prefrontal cortex (dmPFC; *i.e.*, paracingulate gyrus) during non-reinforced CS+ trials compared to CS− trials. Unexpectedly, we also observed that right S1 had negative functional connectivity with early visual sensory regions during non-reinforced CS+ trials compared to CS− trials, which is interesting given that the task did not involve a strong visual component.

**Table 1:** Significant whole-brain clusters showing connectivity with the right S1 seed region. The regions of local maxima peak is derived from sub-regions within the larger cluster based on the Harvard-Oxford atlas in FSL.

Cluster #	Cluster k	Region of Local Maxima	*Z*	*p*-Val	*H*	MNI		
						*x*	*y*	*z*
**CS+ > CS−**								
1	1369	Superior/Middle Frontal Gyrus (dlPFC)	3.41	<0.001	R	34	46	28
		Paracingulate	3.06	<0.005	R	6	52	10
		Middle Frontal Gyrus	3.02	<0.005	R	38	30	42
2	577	Superior/Middle Frontal Gyrus (dlPFC)	3.89	<0.001	L	−24	44	36
**CS− > CS+**								
1	660	Occipital pole	3.80	<0.001	L	−2	−98	10
		Lingual gyrus	3.18	<0.001	R	10	−76	−6

MNI = Montreal Neurological Institute; Cluster # = the nominal cluster number assigned based on cluster size moving from the largest to smallest cluster; Cluster k = number of contiguous voxels; *Z* = *z*-score; *p*-val = *p*-value derived from *z*-score; *H* = hemisphere; L = left; R = right.

### 3.6. Whole-Brain Analysis

To provide a broader context for our individual difference analyses we performed a whole-brain analysis of CS+ *versus* CS− (see Table 2). As expected, this revealed greater activity for CS+ compared to CS− trials in the putative fear network, including insula, dorsal anterior cingulate cortex, and thalamus. It also revealed a similar pattern of activity in auditory cortices, and a larger cluster that included parts of the right motor and somatosensory cortices.

**Table 2:** Voxelwise analysis for tone only model with peak MNI coordinates. The regions of local maxima peak is derived from sub-regions within the larger cluster based on the Harvard-Oxford atlas in FSL.

Cluster #	Cluster k	Region of Local Maxima	*Z*	*p*-Val	*H*	MNI		
						*x*	*y*	*z*
**CS+ > CS−**								
1	9034	Frontal Orbital Cortex	5.10	<0.001	R	38	24	−6
		Insula	4.72	<0.001	R	38	18	−6
		Caudate	4.57	<0.001	R	10	12	2
		Caudate	4.28	<0.001	L	−8	8	2
		Heschl’s Gyrus	3.79	<0.001	R	50	−20	10
		Temporal Pole	3.55	<0.001	R	50	16	−12
		Thalamus	3.49	<0.001	R	8	−16	6
		Angular Gyrus	3.36	<0.001	R	54	−50	18
		Putamen	3.21	<0.001	R	32	−14	2
		Inferior Frontal Gyrus	3.19	<0.001	R	54	16	8
		Thalamus	3.05	<0.005	L	−12	−18	6
		Middle Temporal Gyrus	2.79	<0.005	R	68	−34	−12
		Supramarginal Gyrus	2.71	<0.005	R	64	−24	28
		Putamen	2.48	<0.01	L	−18	6	−2
2	4936	Anterior Cingulate Cortex	4.37	<0.001		4	6	38
		Superior Parietal Lobule	4.16	<0.001	R	32	−44	68
		Posterior Cingulate Cortex	3.69	<0.001		6	−22	44
		Precentral Gyrus	3.25	<0.001	R	46	−6	54
		Paracingulate	3.18	<0.001		6	40	26
		Supplementary Motor Cortex	3.07	<0.005	R	6	−6	58
		Supplementary Motor Cortex	2.42	<0.01	L	−2	4	58
		Superior Frontal Gyrus	2.41	<0.01	R	4	28	52
3	1198	Frontal Orbital Cortex	4.36	<0.001	L	−32	24	−8
		Insula	3.32	<0.001	L	−40	10	−6
		Temporal Pole	2.69	<0.005	L	−54	8	−6
**CS− > CS+**								
1	2450	Middle Frontal Gyrus	3.12	<0.001	L	−34	24	48
		Subcallosal Cortex	2.62	<0.005	L	−6	24	−12
		Superior Frontal Gyrus	2.52	<0.01	L	−12	28	58
		Superior Frontal Gyrus	2.35	<0.01	R	22	26	58
2	1362	Temporal Fusiform Cortex	3.36	<0.001	L	−36	−40	−22
		Inferior Temporal Gyrus	2.88	<0.005	L	−50	−56	−14
		Hippocampus	2.73	<0.005	L	−28	−38	−4
		Parahippocampal Gyrus	2.62	<0.005	L	−22	−36	−18
		Temporal Occipital Fusiform	2.31	<0.05	L	−38	−50	−20
3	886	Postcentral Gyrus	3.59	<0.001	L	−54	−8	26
4	877	Middle Frontal Gyrus	3.24	<0.001	R	34	30	44
		Frontal Pole	3.06	<0.005	R	22	44	46
		Frontal Pole	2.46	<0.01	L	−10	44	48

MNI = Montreal Neurological Institute; Cluster # = the nominal cluster number assigned based on cluster size moving from the largest to smallest cluster; Cluster k = number of contiguous voxels; *Z* = *z*-score; *p*-val = *p*-value derived from *z*-score; *H* = hemisphere; L = left; R = right.

## 4. Discussion

The purpose of the present study was to determine the significance of anticipatory activity in the primary sensory cortex corresponding to an unconditioned stimulus (*i.e.*, US-related brain activity). Specifically, we first sought to determine how such activation relates to individual differences in anxiety. Second, we aimed to determine the degree to which US-related brain activity participates in a process of multisensory integration involved in associative learning and fear conditioning. To address our aims we used a differential trace conditioning paradigm with partial reinforcement in which one auditory tone was paired with a mild shock to the left hand while a second tone was never paired with shock. Consistent with our first prediction we found that trait anxiety was positively correlated with individual differences in fear-induced right S1 activity (*i.e.*, CS+ > CS−), and not left S1 activity, on non-reinforced trials, as only the left hand received the mild shock. Furthermore, consistent with our second prediction, greater right S1 activity on non-reinforced CS+ *versus* CS− trials was positively correlated with regions of the fear network, particularly the amygdala and the primary auditory cortex. We followed up on this observation with a mediation analysis to elucidate how activity in right S1, A1 and the amygdala were related to each other in terms of a functional network. This revealed that activity in right S1 is positively related to A1 activity directly (*i.e.*, *c’* in Figure 2 after controlling for mediation by the amygdala) and positively related to A1 activity indirectly as mediated by the amygdala (*i.e.*, *abs* in Figure 2). Lastly, we performed a seed-based functional connectivity analysis to examine the trial-by-trial neural correlates of right S1 activity. Consistent with our prediction that greater CS+ activity on non-reinforced trials, compared to CS− trials, in right S1 would correlate with activity in regions involved in higher-order cognitive processing, we observed greater functional connectivity between right S1 and dlPFC/dorsomedial prefrontal cortex (dmPFC).

### 4.1. Interactions between Anxiety and Sensory Processing

Along with early findings in the literature, our results suggest that trait anxiety is associated with greater predictive activity in the primary sensory cortex corresponding to the US. Previous observations in the human neuroimaging literature have found increased activity in US-related brain regions on non-reinforced trials [28], particularly early on in associative learning [18]. In the present study, greater anticipatory threat-related activity in right S1 on non-reinforced trials was positively correlated with individual differences in anxiety. The specificity of this observation was confirmed by the fact that in the left S1, which responds to the side of the body that was never shocked, there was no significant relationship between neural activity on non-reinforced CS+ *versus* CS− conditions and trait anxiety. This finding is consistent with behavioral reports indicating that anxiety is positively related to the expectation of negative reinforcement [4,51]. In a recent example, Boddez *et al.* [52] used a blocking paradigm to demonstrate that individuals high in trait anxiety had higher expectations of negative reinforcement during presentations of the blocked CS+. In another study, Robinson *et al.* [53] demonstrated that anxiety induction enhanced the processing of threat-related information. These behavioral findings are also consistent with a recent fMRI observation that anxiety modulated the impact of aversive unconditioned stimuli on primary sensory cortex activity [54], though this involved aversive trials per se rather than anticipatory threat trials. Nevertheless, these findings speak to the interaction between anxiety and sensory processing. An alternative interpretation is that rather than a prediction or conditioned expectancy, the S1 activity we observed is actually a prediction error. That is, S1 activity is being triggered by the absence of shock (the violation of an expectation) [55]. Unfortunately, the current design does not allow us to disentangle these two possibilities, which future studies with a longer trace interval could [56]. However, given the multisensory integration results discussed below, in which S1 activity is integrated into a fear conditioned neural network, we interpret our findings with reference to anticipatory, or expected, threat. If the observed S1 activity instead related to a positive appetitive prediction error (*i.e.*, no punishment when it was expected), we would anticipate no positive correlation between S1 and the fear conditioned network.

Our findings also indicate that early sensory regions are not simply responding to physical stimulation. Instead, there appears to be a synergistic relationship between anxiety, and the sensory processing and anticipation of threat-related stimuli. In addition to the previously discussed findings regarding US-related brain activity, we also observed greater activity in the primary sensory cortex corresponding to the conditioned stimuli, A1, on CS+ compared to CS− trials. This observation is consistent with a number of fear conditioning studies using conditioned stimuli in the visual [18,19], the auditory [20,21], and the olfactory [22] domains.

### 4.2. Fear-Conditioning and the Importance of Multisensory Integration

In addition to the relevance of anticipatory activity in the primary sensory cortex of the UC (*i.e.*, right S1), multisensory integration is one important facet of associative learning that has been often ignored in recent studies of fear conditioning in fMRI. The current study identified that individual differences in right S1 activity covary along with primary auditory cortices (A1) and the amygdala. The interaction of these three regions in an associative network was further confirmed using a mediation analysis demonstrating that the amygdala mediated a significant proportion of the relationship between A1 and right S1.

Along with previous findings, our study suggests that during the acquisition or formation of threat-related associations there is an important process of multisensory integration that varies as a function of individual differences. Although we are aware of no fMRI studies that have investigated a fear-related network in this manner, previous research involving human EEG supports our findings. Miltner *et al.* [27] performed a delay conditioning task in which one of two colors was paired with a mild shock. Notably, they found significant gamma coherence (*i.e.*, functional connectivity) between regions of early visual cortex (*i.e.*, sensory cortex for the CSs) and the somatosensory regions corresponding to the hand that received the shock. Although Miltner *et al.* (1999) used delay conditioning with 100% reinforcement, the present study demonstrates a similar pattern of results using trace conditioning. Moreover, unlike the previous study, which used delay conditioning and analyzed reinforced trials, the current study relied on the analysis of non-reinforced trials due to the slow nature of the BOLD response. This had the added benefit of demonstrating that the processes of multisensory integration in the context of associative learning continues even during quiescent periods of the task. It is also noteworthy that studies carried out in animal models have made similar observations [13]. Additionally, the individual differences we observed in the integration of sensory signals with the amygdala may be akin to activity observed in the fear network during instances of uncertain threat [57], potentially representing the facilitation of contingency learning.

### 4.3. Anxiety and Multisensory Integration

Our findings are also consistent with growing evidence demonstrating the importance of sensory cortices, and their connections with the amygdala, during the processing of threatening information in anxiety [58,59,60,61]. For example, Doehrman *et al.*, (2013) demonstrated that visual cortex activity in patients with social anxiety disorder is a biomarker for predicting cognitive behavioral treatment success. Specifically, those patients with greatest visual cortex activity in response to emotional *versus* neutral stimuli displayed greater symptom reductions following treatment [59]. Similarly, in a large sample of anxious youth, Price *et al.* (2014) found impaired attentional disengagement from threat as indexed by greater activity in the fusiform gyrus of anxious compared to control participants [60]. More recently, a diffusion tensor imaging study found that more robust structural connections between the amygdala and perceptual regions such as the inferior temporal cortex and medial aspect of the somatosensory cortex contribute to higher trait anxiety [62]. Together, findings such as these are indicative of facilitated connections between sensory and affect-related brain areas in anxious individuals, which may serve to perpetuate threat-related attentional biases. Moreover, there is growing interest in the use of attentional retraining of anxious individuals to reverse their attentional biases towards threatening stimuli (e.g., Attention Bias Modification Treatment; ABMT) [63,64]. One possibility is that the success of such interventions relates to the strengthening of neural mechanisms involved in emotion regulation by attention control that are necessary for disrupting connections between sensory regions and the amygdala [58].

### 4.4. The Role of Higher-Order Processes and Related Regions

Our findings indicate that higher-order processes are involved in anticipatory threat-related reactivity. On a trial-by-trial basis we observed greater functional connectivity between right S1 and bilateral dlPFC during presentation of the CS+ *versus* the CS− tone. This observation is consistent with the involvement of higher-order cognitive factors, such as attention, and related brain regions in modulating the CS-US relationship (e.g., [16]). Much research has detailed the contributions of attention and awareness to the strength of the contingency formation between the CS+ and the US. For example, while contingency awareness in delay conditioning, in which the CS and US temporally overlap, does not appear to be necessary for CR acquisition in all cases [2,3,65], its presence is associated with heightened or more persistent conditioned responding [3,66]. In trace conditioning, where an interval typically lasting a few seconds is present in between the termination of the CS and the commencement of the US, attention and awareness appear necessary for accurate fear conditioning [2,3,10]. Moreover, the dlPFC appears to be one region whose activity correlates with the CS-US contingency awareness of participants in both delay and trace conditioning [15]. Such findings emphasize the importance of higher-order processes in associative learning, particularly as learning demands becomes more complex (*i.e.*, moving beyond single-cue conditioning). We speculate that in the context of the current study, dlPFC is involved in the generation of anticipatory activity via attention-related mechanisms in the neural regions that respond to the US (*i.e.*, the mild shock). Such mechanisms might also include more elaborative processes such as mental imagery related to the expectation of somatosensation or physical threat more generally (e.g., [67,68]).

The functional connectivity analysis also revealed greater connectivity between right S1 and right dorsomedial prefrontal cortex. This may reflect the role of dmPFC in the generation of affective signals via connections with the amygdala [69,70] after the integration of predictive threat signals from sensory cortices. In addition, dmPFC is also associated with the predictive coding of aversive events [71]. For example, Dunsmoor *et al.* [72] found that dmPFC activity linearly increased along with the predictive strength of conditioned stimuli. Alternatively, aspects of the dmPFC are implicated in cognitive control processes such as conflict monitoring [73,74,75], and the dmPFC appears to participate in such processes as part of a larger network responsible for cognitive control that also includes dlPFC (e.g., [76,77,78]). Together with the observation of greater right S1 connectivity with bilateral dlPFC, the dmPFC involvement is most likely related to its role in facilitating the integration of affective signals from the amygdala and its participation in conflict monitoring in relation to predicting threat relevance.

### 4.5. Limitations

The current study did not explicitly ask participants the degree to which they anticipated the shock or their perception of the tone-shock contingency ratio. However, we did observe significantly greater SCR activity for the CS+ *versus* the CS−, which is consistent with previous research measuring anticipatory reactivity when using perceptible conditioned stimuli [2,3,4]. Future research is needed to determine whether the patterns of neural activity observed in the present study would differ if participants were required to make explicit judgments of US expectancy. An additional limitation of the study is that there were twice as many CS+ trials (10 reinforced, 10 non-reinforced) as CS− trials. This was necessary to ensure that there were an equal number of trials included in the two conditions of interest (*i.e.*, the CS+ non-reinforced trials *versus* the CS− trials). Lastly, given the use of trace conditioning, it is possible that the current findings do not generalize to other forms of associative learning, like delay conditioning. However, as noted previously, previous research using EEG found a similar pattern of multisensory integration using delay conditioning [27].

## 5. Conclusions

The present study demonstrates that during threat-related associative learning, anticipatory US-related brain activity is modulated as a function of trait anxiety. Furthermore, individual differences in the anticipatory US-related sensory cortex response are influenced by a network of brain regions involved in the fear learning process, including the amygdala and the primary sensory regions corresponding to the conditioned stimuli. Finally, trial-by-trial activity in the US-related sensory cortex was modulated by parts of the prefrontal cortex involved in higher-order processes. Future work is needed to elucidate just how robust the influence of higher-order processes, such as attention or imagery, are on the early sensory cortices responsible for responding to aversive stimuli (e.g., [79,80]). It is also unclear how the observations made in the current study directly relate to patients suffering from anxiety-related disorders, such as phobias or post-traumatic stress disorder. Nevertheless, the present findings suggest that a focus on the interactions between anxious personality traits and the integration of multisensory information may further our understanding of how anxiety impacts information processing.
